# Cx26 knockout predisposes the mammary gland to primary mammary tumors in a DMBA-induced mouse model of breast cancer

**DOI:** 10.18632/oncotarget.5953

**Published:** 2015-10-02

**Authors:** Michael K.G. Stewart, John F. Bechberger, Ian Welch, Christian C. Naus, Dale W. Laird

**Affiliations:** ^1^ Department of Physiology and Pharmacology, University of Western Ontario, London, Ontario, Canada; ^2^ Department of Cellular and Physiological Sciences, University of British Columbia, Vancouver, British Columbia, Canada; ^3^ Department of Anatomy and Cell Biology, University of Western Ontario, London, Ontario, Canada; ^4^ Animal Care and Veterinary Services, University of Western Ontario, London, Ontario, Canada

**Keywords:** connexin26, mammary gland, breast cancer, DMBA

## Abstract

Down-regulation of the gap junction protein connexin26 (Cx26) is an early event following breast cancer onset and has led to Cx26 being classically described as a tumor suppressor. Interestingly, mutations in theCx26 gene (*GJB2)* reduce or ablate Cx26 gap junction channel function and are the most common cause of genetic deafness. It is unknown if patients with loss-of-function *GJB2* mutations have a greater susceptibility to breast tumorigenesis or aggressive breast cancer progression. To investigate these possibilities, 7, 12-dimethylbenz[α]anthracene (DMBA)-induced tumor development was evaluated in BLG-Cre; Cx26^fl/fl^ mice expressing Cre under the β-Lactoglobulin promoter (Cre+) compared to Cx26^fl/fl^ controlmice (Cre-) following pituitary isograft driven Cx26 knockout. A significantly increased number of DMBA-treated Cre+ mice developed primary mammary tumors, as well as developed multiple tumors, compared to Cre- mice. Primary tumors of Cre+ mice were of multiple histological subtypes and had similar palpable tumour onset and growth rate compared to tumors from Cre- mice. Lungs were evaluated for evidence of metastases revealing a similar percentage of lung metastases in Cre+ and Cre- mice. Together, our results suggest that loss of Cx26 predisposes the mammary gland to chemically induced mammary tumour formation which may have important implications to patients with *GJB2* mutations.

## INTRODUCTION

Breast cancer is the most frequently diagnosed cancer affecting women in the world [1]. Early detection remains a key factor in patient survivability as the 5 year survival rate for stage 1 breast cancer is over 90% compared to < 30% for stage 4 breast cancer [2-4]. Therefore, the identification of at risk populations may be important for early detection of the disease in order to improve patient survivability [5]. In addition, although much progress is being made to understand signaling pathways in breast cancer, key regulators of breast cancer progression and metastasis remain poorly understood due to the complexity of the disease and the identification of these proteins is critical for the development of targeted therapies and biomarkers [6]. Gap junction proteins remain interesting candidates as down-regulation of gap junctions remains one of the earliest events in tumour progression [7].

Gap junctions are clusters of intercellular channels formed by connexin subunits between adjacent cells allowing for metabolic and ionic signaling in a process known as gap junctional intercellular communication (GJIC) [8]. GJIC has been linked to critical cellular functions; such as proliferation, differentiation and apoptosis, which are frequently dysregulated in cancer [9]. In addition to GJIC, gap junction channel independent functions involving the connexin interactome and hemichannel function have been shown to be linked to cell growth [10-14]. The connexin family in humans consists of 21 genes but only Cx26 and Cx43 are unequivocally expressed in the human breast [15]. Similarly, the rodent mammary gland also expresses Cx43 and Cx26 but also expresses Cx30 and Cx32 that are dynamically regulated throughout mammary gland development [15]. Only Cx26 and Cx43 have classically been described as tumour suppressors in the breast based on loss of expression in many mammary tumor cell lines and the fact that ectopic re-expression of these connexins reverts some tumor cells into a more differentiated phenotype both *in vitro* and *in vivo* [11 12, 16, 17]. However, adding to the complexity of the role of connexins in breast cancer, both Cx43 and Cx26 have also been reported to be upregulated in human tumour biopsies at later stages of tumour progression and may even act as tumour facilitators [18-20]. These perplexing reports highlight the need for additional studies, particularly *in vivo*, to clarify the role of connexins throughout the progression of breast cancer from tumour onset to metastasis. In this pursuit, we recently demonstrated that Cx43 had a critical role in suppressing metastasis to the lungs in a genetically-modified mouse model where Cx43 function was greatly reduced [21]. However, the role of Cx26 in the mammary gland has not been assessed in mice and this may be more important than examining Cx43 as mammary neoplasms typically express markers of luminal epithelial cells [22]. In addition, loss-of-function mutations in the *GJB2* gene that encodes Cx26 are common in society and responsible for over 40% of hereditary deafness and many skin diseases [23]. Importantly, the worldwide prevalence of biallelic *GJB2* related hearing loss accounts for 17.3% of cases [23, 24]. The 35delG mutation is by far the most common and results in the premature truncation of Cx26 and complete systemic loss of channel function, thereby acting like a knockout in the context of gap junction channel activity [23]. Thus, whether this patient cohort is more or less susceptible to breast tumor onset and progression could have profound clinical implications [23]. Therefore, using our previously described genetically-modified mice with conditional knockout of Cx26 expression in the mammary gland, we developed a DMBA-induced mouse model of breast cancer [25]. We hypothesized that low levels of Cx26 within the mammary gland would predispose the mammary gland to the onset of tumors and increase tumour progression and incidence of metastases.

## RESULTS

### Cx26 knockout does not result in spontaneous mammary tumors

**Figure 1:**
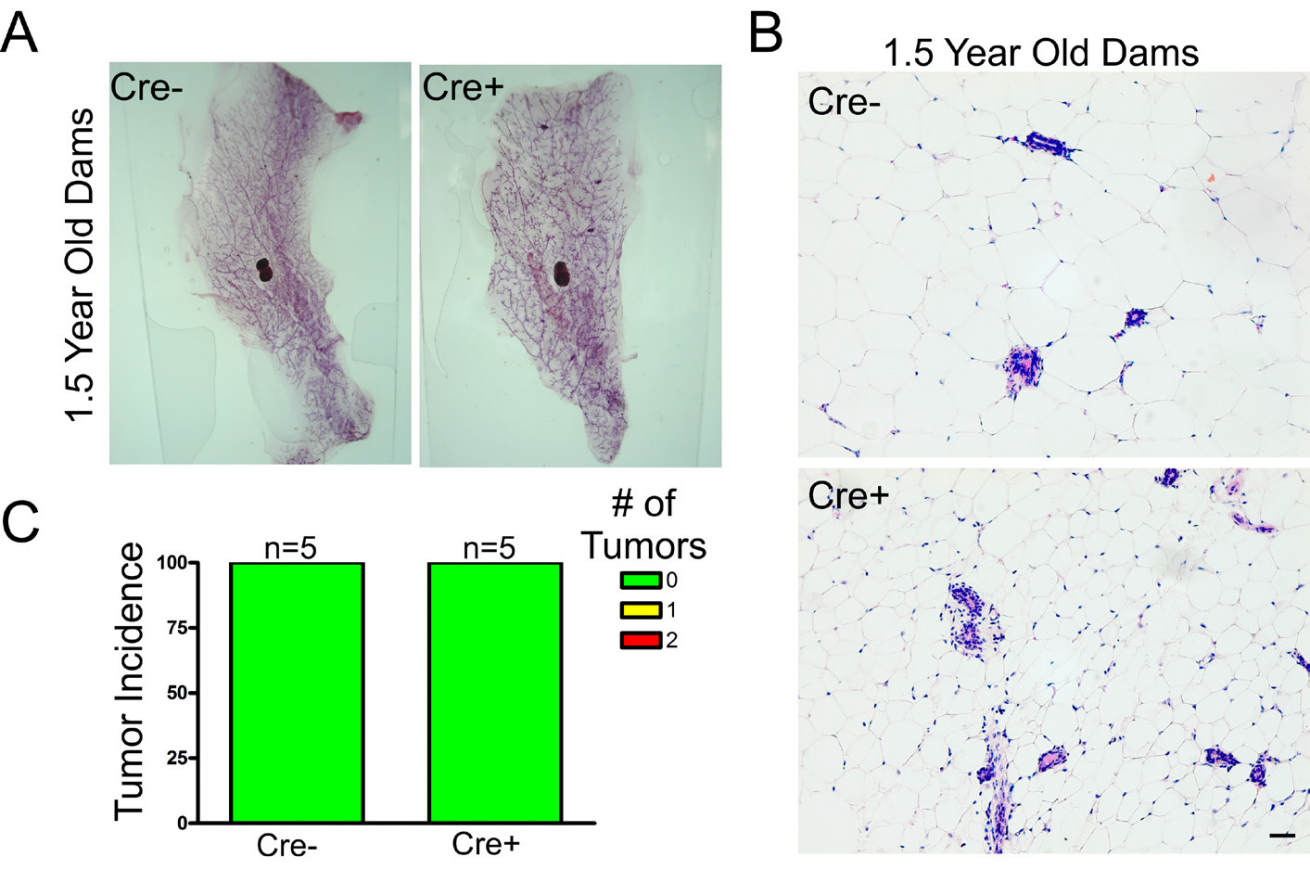
Cx26 knockout does not result in spontaneous mammary tumors **A.**, **B.** Whole mount and hematoxylin and eosin staining revealed normal mammary gland architecture in 1.5 year old Cx26 knockout mice compared to control mice that have undergone at least 2 pregnancies. Scale bars = 50 μm. **C.** Quantification of primary tumour incidence revealed no macroscopic or microscopic tumors in Cx26 knockout mice compared to control mice. N=5.

To evaluate whether conditional Cx26 knockout mice spontaneously developed primary tumors in the mammary gland, five Cx26 knockout and wild-type mice were monitored for 1.5 years before being sacrificed and evaluated for evidence of tumour formation. All mice had at least 2 pregnancies which acted to drive Cx26 knockout as we have previously described [25]. Whole mount and histological evaluation showed no evidence of primary tumors suggesting that a reduction in Cx26 alone is not sufficient to predispose mammary glands to tumour formation (Figure 1).

### DMBA-treated Cx26 knockout mice have greater primary tumour burden compared to control mice but develop mammary tumors with similar growth characteristics

**Figure 2:**
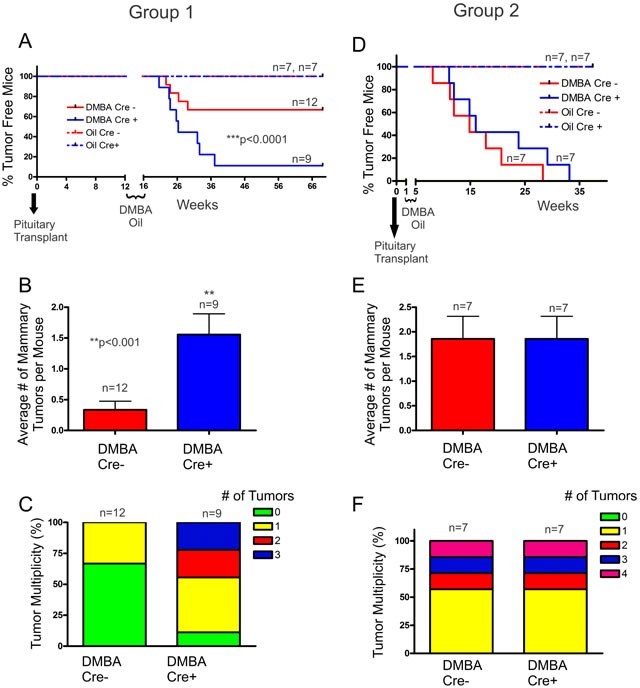
DMBA-treated Cx26 knockout mice developed significantly greater tumor burden **A.** Cx26 knockout mice had a significantly lower number of DMBA-treated Cre+ mice that remained tumour free, a significant increase in the average number of mammary tumors per mouse **B.** and a greater frequency of developing multiple tumors **C.**, compared to control mice. **D.** DMBA-treated Cre+ mice in which DMBA treatment occurred prior to Cx26 knockout had similar tumour burden, average number of mammary tumors per mouse **E.** and a similar frequency of developing multiple tumors **F.** compared to control mice. For B and E, bars represent means ± SEM.

As our Cx26 knockout mice do not develop spontaneous mammary tumors, we used a chemically-induced strategy where the carcinogen DMBA was used in combination with pituitary isografts to induce Cx26 knockout. Following pituitary transplant, Cre- and Cre+ mice were treated with DMBA/oil either after (Group 1) or before (Group 2) Cx26 knockout, and mice were evaluated for palpable tumour onset and number of tumors. No oil treated mice from either group developed mammary gland tumors (Figure 2A, 2D). Interestingly, when comparing Cre- and Cre+ mice in which 3 months had passed before Cx26 knockout, DMBA-treated Cre+ mice had a significantly (*p* < 0.0001, logrank test) lower number of mice that remained tumour free compared to Cre- mice over the duration of the experiment (Figure 2A). This corresponded to 89% of Cre+ mice developing tumors (8/9 Cre+) compared to only 33% of control mice (4/12 Cre-). Importantly, Cre+ mice had a significantly (*p* < 0.001, Student's unpaired t-test) greater average number of mammary tumors per mouse at the end of the experiment compared to Cre- mice; in which half (4/8) of Cre+ mice that developed tumors presented with multiple tumors while Cre- mice only ever developed one tumour over the course of the experiment (Figure 2B, 2C). Alternatively, when DMBA-treatment occurred 1 week following pituitary transplant (and thus no Cx26 knockout) no difference was observed in the number of mice that remained tumour free in which all mice of both groups developed palpable mammary tumors (Figure 2D). In addition, no difference was observed in the average number of mammary tumors per mouse between Cre+ and Cre- mice at the end of the experiment in which 43% (3/7) of both DMBA-treated Cre+ and Cre- mice developed multiple mammary tumors suggesting similar tumor multiplicity (Figure 2E, 2F). Therefore, our results suggest that loss of Cx26 within the mammary gland prior to DMBA-treatment predisposed the mammary gland for increased tumour burden compared to control mice.

**Figure 3:**
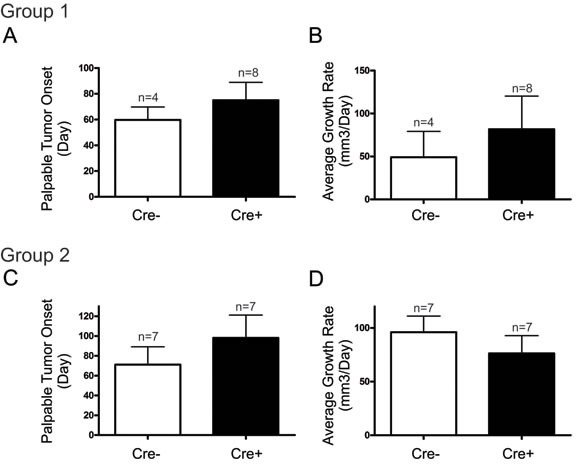
Palpable mammary tumour onset and growth rate is similar in DMBA-treated Cx26 knockout and control mice Mammary tumors from Cre+ mice, both DMBA-treated 3 months **A.**, **B.**, or 1 week **C.**, **D.** after pituitary transplant, had similar primary tumour onset and average growth rate compared to those from Cre - mice. Bars represent means ± SEM.

We next assessed whether the day of palpable tumour onset was earlier in Cx26 knockout mice following the end of DMBA treatment. Mammary tumors from Cre+ mice treated with DMBA following Cx26 knockout had similar palpable tumour onset (75±14 days) compared to Cre- mice (60±10 days) suggesting that despite an increased frequency of developing mammary tumors, tumors arose at comparable times in Cre+ and Cre- mice (Figure 3A). Similarly, palpable tumor onset in mice treated with DMBA 1 week following pituitary transplant was non-significantly different in Cre+ (98±23 days) compared to Cre- mice (71±18 days) (Figure 3C). Taken together, knockout of Cx26 does not appear to increase the day of onset of chemically-induced mammary tumors.

Once the largest mammary tumors reached ~1cm^3^ or ~1 year after DMBA treatment, mice were sacrificed and tissue was collected. Tumour volume of the largest tumour divided by the number of days since palpable tumour onset was used to calculate the average tumour growth rate. Comparing mice treated with DMBA 3 months after pituitary isografts, the average tumour growth rate were non-significantly different between Cre- (49±30 mm^3^/day) and Cre+ (81±39 mm^3^/day) mice suggesting that Cx26 knockout prior to mammary tumour onset did not predispose the gland to primary tumors with increased growth rate (Figure 3B). Similarly, both Cre- (96±16 mm^3^/day) and Cre+ mice (76±17 mm^3^/day) treated with DMBA 1 week after pituitary isografts developed tumors with non-significantly different average tumour growth rates (Figure 3D). Therefore, knockout of Cx26 does not appear to predispose the mammary gland to tumors with increased growth rate when Cx26 knockout occurs either before or after DMBA treatment.

### Cx26 knockout and control mice develop primary mammary tumors of multiple histological subtypes expressing markers of both luminal and myoepithelial cells

**Figure 4:**
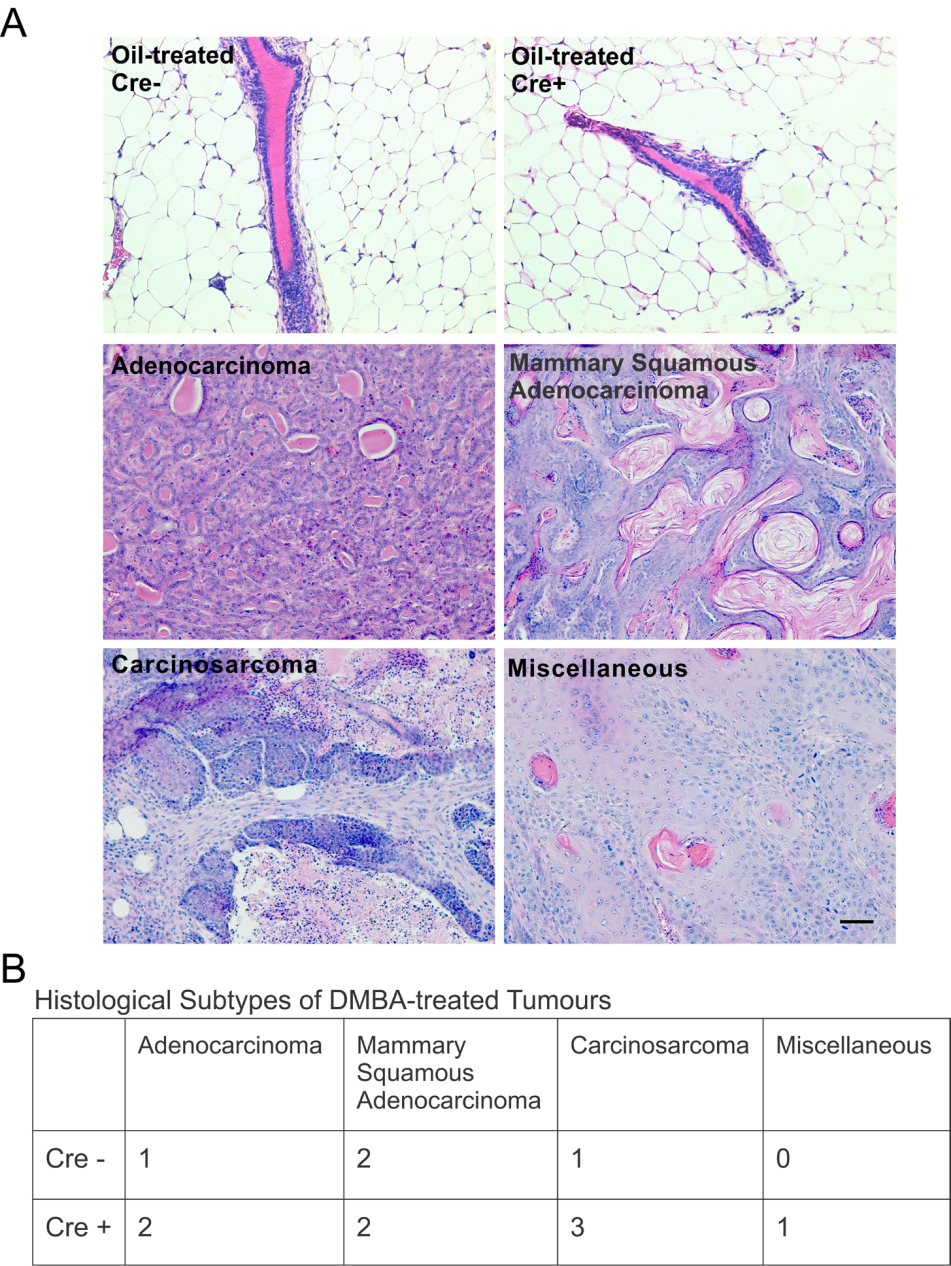
Cx26 knockout mice develop tumors of multiple histological subtypes similar to control mice **A.** Hematoxylin and eosin stained sections evaluated for histological subtypes of breast cancer revealed multiple tumour subtypes for both Cre+ and Cre- mice treated with DMBA 12 weeks after pituitary transplant but no evidence of mammary tumors in oil-treated mice. Scale bar =50 μm. **B.** Table lists the number of mammary tumors per mammary histological subtype for Cre- and Cre+ mice.

As differences in the frequency of mammary tumors arose only between Cre- and Cre+ mice treated with DMBA after Cx26 knockout, we decided to further characterize samples from Group 1 to assess whether Cx26 knockout prior to DMBA treatment predisposed the mammary gland to a specific mammary tumour histological subtype. H&E stained sections of mammary glands from oil-treated Cre- and Cre+ mice revealed normal tissue histology in which typical epithelial ducts were found embedded within an adipose rich mammary fat pad (Figure 4A). Mammary tumour sections stained with H&E from Cre- and Cre+ mice were characterized into either mammary adenocarcinoma, adenosquamous carcinoma, carcinosarcoma or miscellaneous subtypes (Figure 4A) revealing that mammary tumors from Cre+ mice developed into tumors from multiple histological subtypes similar to mammary tumors from Cre- mice (Figure 4B). Therefore, our results suggest that knockout of Cx26 within the mammary gland prior to DMBA-treatment did not predispose the mammary gland to mammary tumors of a single histological subtype.

**Figure 5:**
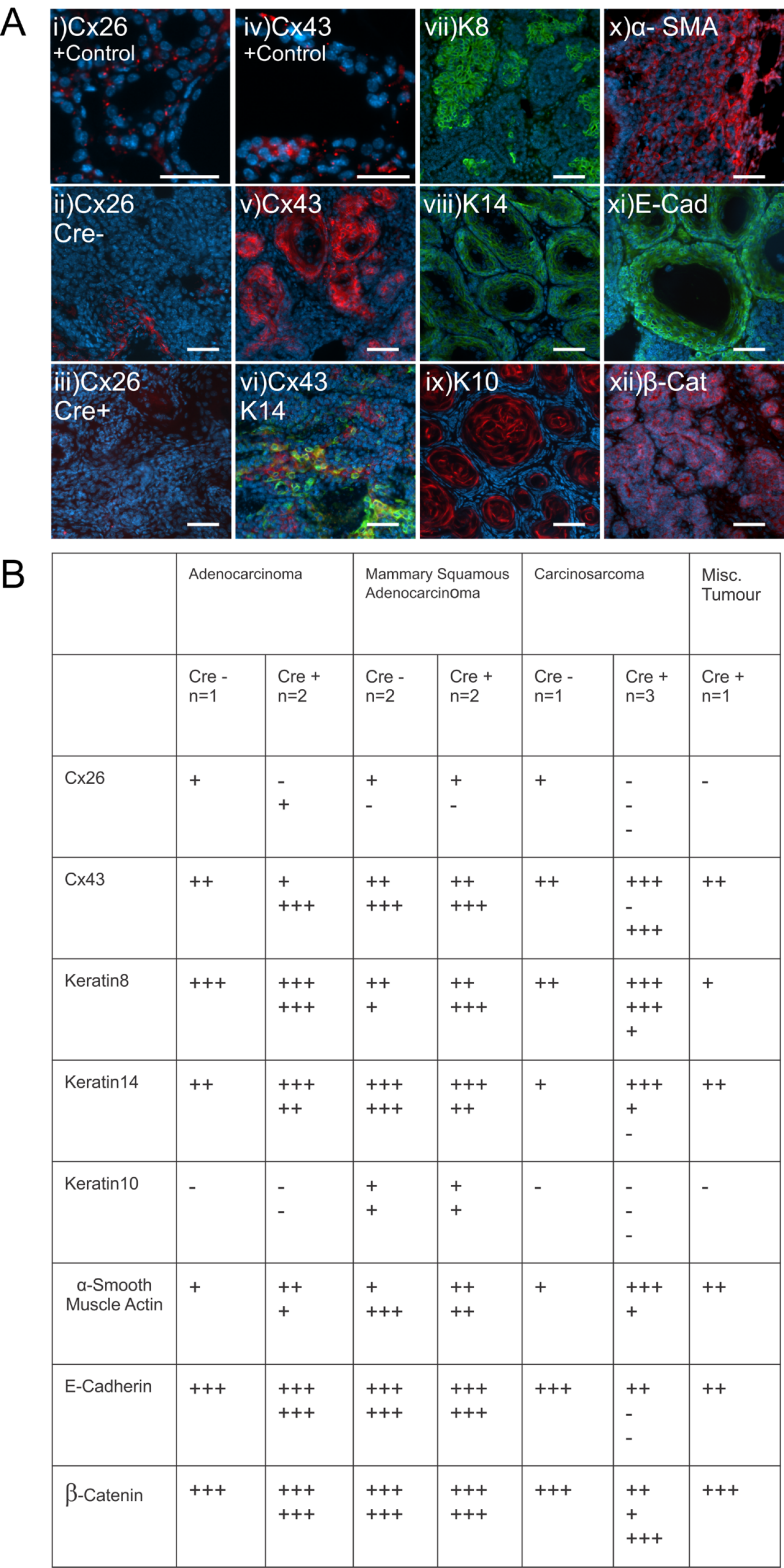
Mammary tumors from Cx26 knockout mice express similar epithelial protein markers as control mice **A.** Representative images of paraffin-embedded primary mammary tumor sections immunolabelled with luminal and myoepithelial markers that included; Cx26 (i, ii, iii, red), Cx43 (iv, v, vi, red), keratin 14 (vi, viii green), keratin 8 (vii, green), keratin 10 (ix, red), α-smooth muscle actin (x, red), E-cadherin (xi, green) and β-catenin (xii, red). Hoechst denotes nuclei. Scale bars=50 μm. **B.** Table indicates relative number of cells that are positive for the luminal and myoepithelial markers based on immunofluorescent labelling. +++50-100%, ++11-49%, +1-10%, −0%.

In order to further assess primary mammary tumors from mice in which Cx26 knockout occurred prior to DMBA treatment, mammary tumors were immunolabelled with a variety of connexin, luminal and myoepithelial markers (Figure 5A) and the percentage of cells expressing the markers were recorded (Figure 5B). Immunofluorescent analysis of Cx26 revealed little to no evidence of Cx26 labelling in tumors from Cre+ mice unlike the lactating mammary gland which acted as a positive control (Figure 5Ai, 5Aiii, 5B). Interestingly, little to no evidence of Cx26 labelling was also observed in tumors from Cre- mice suggesting that Cx26 was also down-regulated in mammary tumors that did not express the Cre transgene (Figure 5Aii, 5B). In addition, mammary tumors immunolabelled for the expression of Cx43 revealed no overt difference in the level of Cx43 in the mammary tumors of both Cre+ and Cre- mice although the expression appeared diffuse and intracellular compared to the more punctate pattern seen in the lactating mammary gland (Figure 5Aiv, 5Av, 5B). Cx43 was also expressed in carcinoma cells that did not always co-localize with the myoepithelial marker keratin14 (Figure 5Avi, 5B). To assess for the expression of luminal and myoepithelial markers, when tumors were immunolabelled for the luminal markers keratin 8, E-cadherin and β-catenin and the myoepithelial markers keratin14 and α-smooth muscle actin, no distinguishable differences were observed between Cre- and Cre+ mice (Figure 5Avii, 5Aviii, 5Ax, 5Axi, 5Axii, 5B). Finally, only adenosquamous carcinoma tumors from both Cre- and Cre+ mice labelled positively for the skin marker keratin 10 typical of the epidermoid differentiation of these tumors (Figure 5Aix). Taken together, mammary tumors from mice in which Cx26 was knocked down prior to DMBA treatment express similar luminal and myoepithelial markers to mammary tumors from control mice.

### Cx26 knockout and control mice exhibit similar levels of metastasis to the lungs

**Figure 6:**
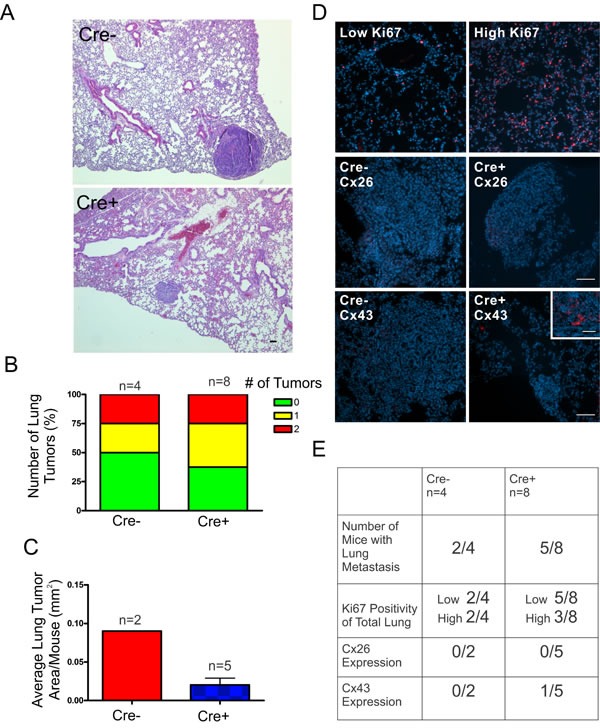
DMBA-treated Cx26 knockout mice exhibit similar incidence of metastases to the lungs **A.**, **B.** Hematoxylin and eosin stained lung sections were evaluated for evidence of lung tumors revealing a similar proportion of mice that developed lung tumors in Cre+ and Cre- mice. **C.** Evaluation of average lung tumor area per mouse between Cre+ and Cre- mice revealed the likelihood of Cre- mice having larger lung tumor areas compared to Cre+ mice. **D.** Representative images of paraffin-embedded lung sections immunolabelled for Ki67 (Red), Cx26 (Red) and Cx43 (Red) revealed a similar percentage of lung tissue expressing high levels of Ki67 positivity between Cre- and Cre+ mice and tumors mostly negative, but not always (insert), for Cx26 and Cx43 expression. Hoechst denotes Nuclei. Scale bars = 50 μm. **E.** Quantification of Ki67, Cx26 and Cx43 immunofluorescent analysis.

Following our evaluation of the primary tumors, we assessed the lung, a common site of metastasis in DMBA-induced mammary tumors, for signs of disseminated disease [21]. Hematoxylin and eosin stained sections of lung tissue revealed evidence of metastasis to the lungs (Figure 6A) in which a similar proportion of mice from Cre+ (50%) and Cre- mice (63%) developed tumors in the lung (Figure 6B). Although only 2 DMBA-treated Cre- mice developed lung tumors, the average lung tumour area per mouse was calculated revealing a likelihood of larger average lung tumour area in Cre- mice (0.09 mm^2^) compared to Cre+ mice (0.02 ± 0.009 mm^2^) (Figure 6C). To evaluate whether lung tissue of Cre- and Cre+ mice had greater evidence of cancer cell infiltration, lung tissue was immunolabelled with Ki67, a marker of proliferating cells, and separated into high (> 50%) and low (< 50%) groups revealing a similar number of mice with high levels of Ki67 staining in Cre- (50%) and Cre+ mice (38%) (Figure 6D, 6E). Lung tumors immunolabelled for Cx26 and Cx43 to evaluate if connexin expression changes between primary and metastatic tumors revealed mostly the absent expression of both Cx26 and Cx43 in Cre- (Cx26: 0/2, Cx43: 0/2) and Cre+ (Cx26: 0/5, Cx43: 1/5) mice suggesting that connexins are not upregulated during metastatic progression (Figure 6D, 6E).

## DISCUSSION

Our study's aims were two-fold; first, to evaluate whether the organ-specific loss of Cx26 predisposed the mammary gland to developing mammary tumors *in vivo*. Secondly, to evaluate if loss of Cx26 in primary mammary tumors led to altered progression and aggressiveness of the disease. In order to evaluate these aims, we developed the first chemically-induced conditionally ablated mouse model of breast cancer to assess the role of Cx26. We demonstrated that knockout of Cx26 prior to tumour induction by DMBA treatment increased the susceptibility of mice to primary mammary tumors but that this increase in the frequency of breast tumour onset was not associated with increased progression of the disease.

### A model to investigate the role of Cx26 in mammary tumorigenesis *in vivo*

Evaluating the *in vivo* role of Cx26 in breast cancer is complicated by the fact that Cx26^−/−^ mice die embryonically due to defects in placenta rendering them unusable for this kind of study [26]. As a result, we used our previously characterized mammary gland specific knockout mouse model of Cx26, in which ~70% knockout of Cx26 was observed in the mammary gland driven by Cre-mediated deletion under the BLG promoter following the onset of lactation [25]. We observed no evidence of spontaneous mammary tumors or abnormal histology in 1.5 year old dams that have undergone at least 2 pregnancies which is in agreement with results from mammary gland specific deletion of Cx26 using similar Cre-loxP strategies under the mouse mammary tumour virus and whey acidic protein promoters [27]. Therefore, it appears that the loss of Cx26 is not sufficient for initiating tumour onset which requires additional genetic insults.

In order to promote additional genetic mutations in Cx26 knockout mice, we used the chemical carcinogen DMBA that preferentially promotes the induction of mammary tumors and paired this with the use of pituitary isografts to drive BLG promoter activity [28]. The combination of pituitary isografts and DMBA have been frequently used to drive mammary tumour development through hormonal and chemical carcinogenesis [29, 30]. This approach was used by Wang et al. where pituitary driven activation of the BLG promoter was used to evaluate deregulated Pak1 activity in murine mammary tumorigenesis [31]. Importantly, we observed a clear reduction in the levels of Cx26 when paired non-tumorigenic mammary glands from all Cre+ mice were compared to Cre- mice at the time of sacrifice indicating that the pituitary isografts were effectively driving the knockout of Cx26, and more notably, that Cx26 knockout persisted throughout the length of the experiment. Therefore, using this strategy we evaluated whether the loss of Cx26 prior to these additional genetic alterations would promote tumour incidence.

### Loss of Cx26 promotes tumour onset in chemically-induced mammary tumorigenesis

Most studies to date suggest a tumour suppressive role for Cx26 early in breast cancer progression based on evidence that Cx26 is frequently absent or down-regulated in human breast cancer cell lines or human primary tumors [17, 19, 32]. In agreement, we observed an increase in primary tumour incidence and tumour multiplicity when Cx26 was knocked down prior to DMBA treatments suggesting that expression of Cx26 acts in the context of a tumour suppressor and protects the mammary gland to primary mammary tumour onset. Somewhat unexpectedly, when DMBA-treatment occurred only one week after the pituitary transplant, a similar tumor burden was observed in Cx26 knockout and control mice. While the pituitary isograft is necessary for Cx26 knockout, it serves a dual purpose as continual hormonal secretion of prolactin, which importantly acts on the ovary to induce synthesis and secretion of progesterone and estrogen, which together promote a greater frequency of chemical carcinogen induced mammary tumors [33]. As a result, the potential tumour suppressive effect of Cx26 may be masked by the pro-tumorigenic effects of hormones secreted from the surgically placed pituitary. Three lines of evidence support this; first, all mice treated with DMBA one week following pituitary transplant developed mammary tumors suggesting hormones secreted or stimulated by pituitary isografts were promoting tumor onset. Secondly, we observed an increase in the frequency of tumor incidence in Cre- mice when the pituitary isografts occurred only one week before DMBA treatment compared to Cre- mice in which the same procedure occurred 12 weeks prior to DMBA treatment. The pituitary driven increase in chemically-induced mammary tumors is the result of increasing the proliferation of epithelial cells of the mammary gland although this appears to be time dependent as the increase in the mitotic index begins to fall after 5 weeks as the levels of estrogen and progesterone drop [34]. Ultimately, differences in the frequency of mammary tumors between mice treated with DMBA one week or 12 weeks after pituitary transplant may be explained by increased ovarian hormonal stimulation by pituitaries transplanted closer to the time of DMBA treatment limiting our assessment of Cx26 in mice treated with DMBA one week following pituitary isograft. Finally, a precedent exists that hormonal influence may override any tumour suppressive effects of Cx26 expression as stably transfected Cx26 expressing MCF7 cells, with strong growth suppressing effects *in vitro,* lacked growth suppressing effects *in vivo* potentially as a result of the pro-tumorigenic effects of 17β estradiol pellets [35]. Taken together, it appears likely that the tumour suppressive effect of Cx26 may be masked by pro-tumorigenic hormones secreted by the pituitary isograft.

The use of conditionally ablated mice did not help us distinguish whether Cx26 functions through a GJIC-dependent or independent mechanism. However, as Cx26 possesses a very short C-terminal tail, it is more likely that the increase in mammary gland tumor incidence in conditional Cx26 knockout mice are the result of gap junction channel or hemichannel related mechanism and not related to the small Cx26 interactome [36-38]. Interestingly, Cx26-linked growth suppression in Hela cells was associated with redistribution of cAMP and activation of protein kinase A that was GJIC-dependent as expression of the R75Y Cx26 mutant, that disrupts gap junction channels but leaves hemichannel function unchanged, did not suppress HeLa cell growth [39]. While the role of Cx26 hemichannels in the context of the mammary gland is unclear, genetically-modified mice with mammary gland specific expression of the G45E or S17F Cx26 mutants, which differ in their respective gap junction channel and hemichannel function, may provide more clarity on the role of Cx26 hemichannels in breast cancer [40, 41]. Taken together, our data revealed that Cx26 protects the mammary gland from DMBA-induced mammary tumour onset but this protective effect may be masked in hormonally driven tumorigenesis.

### Cx26 knockout prior to DMBA treatment does not affect primary tumor growth or histological subtype

Cx26 may regulate primary tumour cell growth and proliferation, anchorage-independent and contact-dependent growth *in vitro,* as well as reduced tumour sizes *in vivo* when orthotopically injected into nude mice through GJIC- dependent and -independent mechanisms [10-12, 17, 35, 42]. As a result, we hypothesized that knockout of Cx26 would contribute to increased tumour size but this was not the case as the average tumor growth rates were similar in Cre- and Cre+ DMBA-treated mice both before and after Cx26 knockout. However, as Cx26 was absent or down-regulated even in the majority of Cre- tumors, it remains likely that Cx26 is down-regulated in tumors early in primary tumour progression; this is supported by studies suggesting that Cx26 may be, at least in part, methylated to induce down-regulation [16, 43]. Ultimately, as the Cx26 status was similar in tumors from Cre- and Cre+ mice our assessment of the role of Cx26 in primary tumour growth is limited. However, Cx26 and tumour growth may not be as critical in the human context as the majority, but not all [20], of studies agree that Cx26 mRNA or protein expression does not correlate with tumour size or Ki67 status [44-47]. The overall role of connexins in breast tumorigenesis has recently become more complex as one recent study suggests that the previously undetected Cx30, Cx32 and Cx46 may also be expressed in human breast tissue [47].

DMBA-induced mammary tumors are often associated with multiple gene expression profiles giving rise to tumors with varying histological subtypes, particularly squamous carcinomas and adenocarcinomas, with many tumors expressing both luminal and basal cell markers [28, 48, 49]. Others have demonstrated a link between human histological subtypes correlating with specific genetic alterations, such as the inactivation of the *CDH1* gene that encodes E-cadherin and is frequently found in lobular carcinomas of the breast [50]. Importantly, there is some evidence that this correlation exists when modelling tumorigenesis in mice as Derkson et al. showed that mammary specific deletion of E-cadherin and p53 resulted in mammary tumors similar to lobular carcinomas [51]. We aimed to test whether loss of Cx26 would promote a greater propensity of developing mammary tumors of a specific histological subtype. Interestingly, in both tumors from Cre+ and Cre- mice we observed a wide variety of histological subtypes supported by varied expression of luminal and myoepithelial markers. Therefore, this suggests that the loss of Cx26 prior to DMBA treatment does not promote the development of mammary tumors of specific histological subtype, which is in agreement with a lack of correlation in human data of Cx26 expression with any histological subtypes [20]. In addition, a majority of studies document a lack of correlation between Cx26 and estrogen receptor, progesterone receptor and HER2 status in microarray or immunohistochemical analysis of human tumour samples [45, 47, 51], although a couple of exceptions have been reported in the case of progesterone receptor [20] and estrogen receptor status [45]. Importantly, our immunofluorescent assessment of Cx43 revealed a more intracellular and diffuse labelling pattern suggesting a potential reduction in the ability of the mammary tumor cells to undergo GJIC. While this was observed in mammary tumors from both Cre+ and Cre- mice, a potential role for Cx43 in regulating tumors cannot be unequivocally excluded in mice with Cx26 knockout as Cx43 has also been suggested to act as a tumor suppressor [15].

### Cx26 knockout does not promote metastatic dissemination to the lungs

The role of Cx26 in breast cancer metastasis remains much more controversial than that in the primary tumour particularly in studies using human samples. Some suggest that Cx26 expression does not correlate with lymph node positivity [20] or overall survival [45, 47] while others have found that higher expression of Cx26 is associated with poor overall patient survival [53], particularly if Cx26 expression is elevated after chemotherapy [46]. Still others have found that Cx26 is upregulated in lymph node metastases compared to matched primary tumors [19]. While our results are limited by the number of mice that developed potential lung metastases, we found no evidence of Cx26 expression in any lung tumors similar to the primary tumors. Thus, the knockout of Cx26 prior to DMBA treatment did not predispose the mammary gland to an increased frequency of metastases suggesting that Cx26 is not acting as a breast cancer metastasis suppressor. In addition, Cx43 was also reduced compared to primary tumors suggesting that Cx43 is down-regulated as tumors progress towards metastasis consistent with Cx43 as a breast cancer metastasis suppressor [21]. Of note, the average lung tumour area from Cre- mice appeared larger than those from Cre+ mice although the N-values were too low for a statistically assessment. While this finding hints that lung tumors without Cre-mediated deletion of Cx26 may grow larger or establish themselves earlier than those from Cre+ mice, we observed no evidence that Cre- lung tumors upregulated their Cx26 expression. As a consequence, our results do not support either a tumour suppressive or facilitating role for Cx26 similar to finding by Chao et al. who found no correlation between upregulated Cx26 expression from primary tumors and metastasis to the lung in human breast cancer patient samples [52]. Taken together, our results support a tumour suppressive role for Cx26 in the context of primary tumour onset but this does not coincide with more aggressive tumors or more frequent metastases in our chemically-induced model of breast cancer.

### Implications to human disease

To date, *GJB2* gene mutations give rise most notably to syndromic and non-syndromic hearing loss with comparable carrier frequencies to other prevalent genetic diseases such as cystic fibrosis and sickle-cell anemia [23]. In addition to hearing loss, many patients will present with skin diseases including Bart-Pumphrey syndrome, Hystrix-like icthyosis with deafness, Vohwinkel syndrome and Keratitis ichthyosis deafness (KID) [54, 55]. Despite these skin diseases being relatively rare, patients presenting with Vohwinkel syndrome and KID syndrome have also been reported to develop skin tumors [56]. Most intriguing, a review of 61 patients with KID reported that ~10% of patients developed squamous cell carcinoma suggesting that loss of functional Cx26 in the skin predisposes KID syndrome patients to skin cancer [57]. Although it remains unknown whether other tissues or organs that commonly express Cx26 are also susceptible to developing tumors, a single patient presenting with KID syndrome also presented with a primary invasive sccirrhous ductal carcinoma of the breast suggesting that a loss-of-function mutant of Cx26 may also have contributed to the onset of a primary breast tumour in humans similar to our conditional Cx26 knockout mouse model of breast cancer [58]. However, although our results suggest an increased breast cancer risk to patients with loss of function *GJB2* mutations, it is important to note that our mouse model does not recapitulate all Cx26 mutants. Many are reported to have gain-of-functions, particularly those associated with skin diseases, with increased Cx26 hemichannel function in addition to loss of gap junction channel function [24]. As a result, our findings may extend to only a subpopulation of patients with loss-of-function *GJB2* gene mutations. Therefore, we recommend a large epidemiologic study of breast cancer frequency in patients with loss-of-function *GJB2* mutations compared to familial healthy controls. In that over 1% of the general population worldwide are estimated to be carriers of mutant alleles of *GJB2* it remains critical to determine if this population as a whole, or in part, are at an increased risk of developing breast cancer [23].

## MATERIALS AND METHODS

### Mice

All experimental procedures were approved by the Committee on the Ethics of Animal Experiments at the University of Western Ontario and the University of British Columbia following the guidelines of the Canadian Council on Animal Care. To assess how loss of Cx26 would affect primary mammary tumour development, we utilized a conditional knockout mouse where the Cre transgene was under the control of the β-lactoglobulin (BLG) promoter [25]. In order to induce activation of the BLG promoter and subsequent Cx26 knockout, a pituitary isograft was surgically inserted into the renal capsule (SFig 1A) of 6 week old BLG-Cre; Cx26^fl/fl^ (Cre+) similar to that described by others [59, 31] in addition to Cx26^fl/fl^ (Cre-) control mice lacking the Cre transgene. Prolactin and ovarian hormones derived from the pituitary transplant triggers epithelial proliferation and lobuloalveolar differentiation in the mammary gland. The presence of milk proteins following pituitary isograft have been reported between 21-40 days following surgery, in which we observed the presence of milk in the mammary glands approximately 50 days after pituitary implantation [60, 31]. This suggests that the activation of the BLG promoter occurs between 3-7 weeks in parallel with lobuloalveolar development (SFig 1B). As such, in an attempt to induce mammary tumors in mice in which Cx26 knockout had occurred, Cre+ and Cre- mice, in which pituitaries were implanted 12 weeks prior, were treated once a week with the carcinogen 7,12-dimethylbenz(α)anthracene (DMBA) or corn oil for 5 weeks (1mg per 25g) by gavage (STable 1). In addition, a second experiment was performed to act as a control in which 6 week old BLG-Cre; Cx26^fl/fl^ and Cx26^fl/fl^ mice were treated with DMBA or corn oil by gavage only 1 week following pituitary transplant in order to induce mammary tumors in mice prior to Cx26 knockout (STable 2). All mice were subsequently monitored weekly by palpation for evidence of mammary tumour formation. Mice were removed from the study if they presented with other health concerns that included developing lymphoma and/or stomach tumors that required them to be sacrificed (STable 1, 2). Mice were sacrificed when the largest tumors reached a final volume of 1cm^3^. Finally, five BLG-Cre; Cx26^fl/fl^ and Cx26^fl/fl^ mice that had undergone at least 2 pregnancies were monitored for spontaneous mammary tumour formation for 1.5 years in which tissue was collected similar to that described above. All mice were genotyped for the Cre transgene and mammary gland cryosections were immunolabelled at the time of sacrifice for the presence of Cx26 revealing Cx26 knockout even after 1 year of the experiment (SFig 1C).

### Whole Mount

Whole mounts were performed similar to how we have previously described [61]. Briefly, mammary glands were dissected and flattened onto a glass slide before being placed into Carnoy's fixative (100% EtOH, chloroform, glacial acetic acid; 6:3:1) overnight at 4°C. Glands were immersed in 70% ethanol for 15 min and then transferred to descending concentrations of ethanol before being placed in carmine alum stain overnight at room temperature. Glands were put through increasing concentrations of ethanol and into xylene overnight. Glands were then placed in methyl salicylate for long term storage. Images were captured using a numeric camera (Sony Cybershot).

### Hematoxylin and eosin staining

Dissected mammary tumors, mammary glands and lungs were fixed in 10% neutral buffered formalin and embedded in paraffin wax. Sections (6μm) were deparaffinized in xylene (2×5min) and rehydrated in descending concentrations of ethanol (2×100%, 95%, 70%; 5min) before being placed in ddH_2_O (2×3min). Tissues were then stained in 1% Harris's Hematoxylin for 1 min before being washed in tap water and differentiated in acid ethanol. Slides were dipped in 70% ethanol (30sec) and put into alcoholic Eosin (2min) and finally placed back into 70% ethanol (2×30sec). Slides were then dehydrated and mounted using Cytoseal. Images were captured using a Brightfield microscope equipped with a ProgRes C5 camera (Jenoptik) and ProgRes Mac CapturePro 2.7.6 imaging software. Multiple rounds of sectioning and staining were used to evaluate lung histology for evidence of lung metastases. Clusters of nuclei in intimate proximity with bronchioles, typical of bronchus associated lymphatic tissue, were not included in the quantification of lung tumors. Lung tumour area was evaluated by measuring the length and width of lung tumors which were used to determine the area of an ellipse using ImageJ and the area of multiple lung tumors per mouse was averaged (National Institutes of Health, Bethesda, MD).

### Immunofluorescence and microscopy

Paraffin-embedded sections (6 μm) were deparaffinized in xylene and dehydrated in descending concentrations of ethanol before being placed in ddH_2_0. Antigen retrieval was performed using either antigen unmasking solution (Vector Labs; microwaved for 5 min at 80% power) or by immersing slides in sub-boiling 1.8 mM citric acid and 8.2 mM of sodium citrate solution for 10 minutes before placing slides in an additional 0.01M Tris-0.001M EDTA antigen retrieval solution sub-boiling for 20 min. Cryosections were cut with a cryostat (8um) and stored at −80C and were fixed for 15 min in 10% neutral buffered formalin before use. All tissue sections were rinsed in PBS for 5 min and blocked with 3% BSA in PBS with 0.02% Triton X-100. Sections were immunolabelled with the primary antibodies mouse anti-Cx26 (1:100, Invitrogen, 13-8100) or rabbit anti-Cx26 (Cryosections, 1:100, Invitrogen, 51-2800), rabbit anti-Cx43 (1:400 dilution, Sigma, C6219), rabbit anti-keratin8 (1:400, Abcam, ab53280), mouse anti-cytokeratin 14 (1:200, Thermo Scientific, Ms-115-P), and rabbit anti-keratin 10 (1:400, Thermo Scientific, MS 611-P1), mouse anti-E-cadherin (1:400, BD Transduction Laboratories, 610182), mouse anti-β-catenin (1:400, BD Transduction Laboratories, 610154), α-smooth muscle actin (1:400, Sigma, A5228) overnight at 4°C. Sections were washed in PBS and then probed with Alexa Fluor® 555-conjugated anti-rabbit or anti-mouse (1:400 dilution, Molecular Probes, A21425 or A21429) and Alexa Fluor® 488-conjugated anti-rabbit or anti-mouse (1:400 dilution, Molecular Probes, A11008 or A11017) secondary antibodies for 1 hour at room temperature. Hoechst 33342 was used to label the nuclei and slides were mounted with Airvol. Images were captured using a Leica DM IRE2 inverted epifluorescence microscope and Velocity imaging software. Qualitative assessment of 5-10 images throughout mammary tumors were used to determine the percentage of cells expressing epithelial markers and connexins.

### Histological subtyping

Hematoxylin and eosin stained sections were classified according to histological subtype similar to that described by Dunn [62]. Mammary glands were classified into 4 groups; mammary alveolar carcinoma, mammary adenosquamous carcinoma, carcinosarcoma and miscellaneous tumors. Briefly, mammary alveolar carcinomas represented tumors with mainly uniform alveolar structure of glandular epithelial origin. Mammary adenosquamous carcinomas were characterized by tumors that acquired the capacity for epidermoid differentiation. Carcinosarcomas were represented by tumors classified as anaplastic and mainly devoid of distinct morphological differentiation or with significant amounts of spindle cells. Any tumors that presented outside of the previous three groups was considered a miscellaneous tumour. All tumors were evaluated in a blinded fashion.

### Statistical analysis

All statistical tests were performed using Graphpad Prism 4 (v. 4.02). For analysis of the percent of tumour free mice over time curves, a logrank test was applied. Palpable tumour onset, average number of tumors per mouse and average growth rate were evaluated using a two tailed student's unpaired t-test. A p value less than 0.05 was considered significant.

## SUPPLEMENTARY MATERIAL FIGURE AND TABLES



## References

[1] Jemal A, Bray F, Center MM, Ferlay J, Ward E, Forman D (2011). Global cancer statistics. CA Cancer J Clin.

[2] Berry DA, Cronin KA, Plevritis SK, Fryback DG, Clarke L, Zelen M, Mandelblatt J JS, Yakovlev AY, Habbema JD, Feuer EJ, Cancer Intervention, Surveillance Modeling Network (CISNET) Collaborators (2005). Effect of screening and adjuvant therapy on mortality from breast cancer. N Engl J Med.

[3] de Gelder R, Heijnsdijk EA, Fracheboud J, Draisma G, de Koning HJ (2015). The effects of population-based mammography screening starting between age 40 and 50 in the presence of adjuvant systemic therapy. Int J Cancer.

[4] Chen L, Linden HM, Anderson BO, Li CI (2014). Trends in 5-year survival rates among breast cancer patients by hormone receptor status and stage. Breast Cancer Res Treat.

[5] Anothaisintawee T, Wiratkapun C, Lerdsitthichai P, Kasamesup V, Wongwaisayawan S, Srinakarin J, Hirunpat S, Woodtichartpreecha P, Boonlikit S, Teerawattananon Y, Thakkinstian A (2013). Risk factors of breast cancer: a systematic review and meta-analysis. Asia Pac J Public Health.

[6] Eroles P, Bosch A, Perez-Fidalgo JA, Lluch A (2012). Molecular biology in breast cancer: intrinsic subtypes and signaling pathways. Cancer Treat Rev.

[7] Mesnil M (2002). Connexins and cancer. Biol Cell.

[8] Laird DW (2006). Life cycle of connexins in health and disease. Biochem J.

[9] Wei CJ, Xu X, Lo CW (2004). Connexins and Cell Signaling in Development and Disease. Annu. Rev Cell Dev Biol.

[10] Qin H, Shao Q, Thomas T, Kalra J, Alaoui-Jamali M (2003). A, Laird DW. Connexin26 Regulates the Expression of Angiogenesis-Related Genes in Human Breast Tumor Cells by both GJIC-Dependent and -Independent Mechanisms. Cell Commun Adhes.

[11] Qin H, Shao Q, Curtis H, Galipeau J, Belliveau DJ, Wang T, Alaoui-Jamali MA, Laird DW (2002). Retroviral Delivery of Connexin Genes to Human Breast Tumor Cells Inhibits *in vivo* Tumor Growth by a Mechanism that is Independent of Significant Gap Junctional Intercellular Communication. J Biol Chem.

[12] McLachlan E, Shao Q, Wang HL, Langlois S, Laird DW (2006). Connexins Act as Tumor Suppressors in Three-Dimensional Mammary Cell Organoids by Regulating Differentiation and Angiogenesis. Cancer Res.

[13] Zhou JZ, Jiang JX (2014). Gap junction and hemichannel-independent actions of connexins on cell and tissue functions—an update. FEBS Lett.

[14] Evans WH, De Vuyst E, Leybaert L (2006). The gap junction cellular internet: connexin hemichannels enter the signalling limelight. Biochem J.

[15] McLachlan E, Shao Q, Laird DW (2007). Connexins and gap junctions in mammary gland development and breast cancer progression. J Membr Biol.

[16] Lee SW, Tomasetto C, Paul D, Keyomarsi K, Sager R (1992). Transcriptional downregulation of gap-junction proteins blocks junctional communication in human mammary tumor cell lines. J Cell Biol.

[17] Hirschi KK, Xu CE, Tsukamoto T, Sager R (1996). Gap junction genes Cx26 and Cx43 individually suppress the cancer phenotype of human mammary carcinoma cells and restore differentiation potential. Cell Growth Differ.

[18] Jamieson S, Going JJ, D'Arcy R, George WD (1998). Expression of gap junction proteins connexin 26 and connexin 43 in normal human breast and in breast tumours. J Pathol.

[19] Kanczuga-Koda L, Sulkowski S, Lenczewski A, Koda M, Wincewicz A, Baltaziak M, Sulkowska M (2006). Increased expression of connexins 26 and 43 in lymph node metastases of breast cancer. J Clin Pathol.

[20] Naoi Y, Miyoshi Y, Taguchi T, Kim SJ, Arai T, Tamaki Y, Noguchi S (2007). Connexin26 expression is associated with lymphatic vessel invasion and poor prognosis in human breast cancer. Breast Cancer Res Treat.

[21] Plante I, Stewart MK, Barr K, Allan AL, Laird DW (2011). Cx43 suppresses mammary tumor metastasis to the lung in a Cx43 mutant mouse model of human disease. Oncogene.

[22] Visvader JE (2009). Keeping Abreast of the Mammary Epithelial Hierarchy and Breast Tumorigenesis. Genes Dev.

[23] Chan DK, Chang KW (2014). GJB2-associated hearing loss: systematic review of worldwide prevalence, genotype, and auditory phenotype. Laryngoscope.

[24] Lee JR, White TW (2009). Connexin-26 mutations in deafness and skin disease. Expert Rev Mol Med.

[25] Stewart MK, Plante I, Bechberger JF, Naus CC, Laird DW (2014). Mammary gland specific knockdown of the physiological surge in Cx26 during lactation retains normal mammary gland development and function. PLoS One.

[26] Gabriel HD, Jung D, Butzler C, Temme A, Traub O, Winterhager E, Willecke K (1998). Transplacental uptake of glucose is decreased in embryonic lethal connexin26-deficient mice. J Cell Biol.

[27] Bry C, Maass K, Miyoshi K, Willecke K, Ott T, Robinson GW, Hennighausen L (2004). Loss of connexin 26 in mammary epithelium during early but not during late pregnancy results in unscheduled apoptosis and impaired development. Dev Biol.

[28] Currier N, Solomon SE, Demicco EG, Chang DL, Farago M, Ying H, Dominquez I, Sonenshein GE, Cardiff RD, Xiao ZX, Sherr DH, Seldin DC (2005). Oncogenic signaling pathways activated in DMBA-induced mouse mammary tumors. Toxicol Pathol.

[29] Li B, Kittrell FS, Medina D, Rosen JM (1995). Delay of dimethylbenz[a]anthracene-induced mammary tumorigenesis in transgenic mice by apoptosis induced by an unusual mutant p53 protein. Mol Carcinog.

[30] Jerry DJ, Kittrell FS, Kuperwasser C, Laucirica R, Dickinson ES, Bonilla PJ, Butel JS, Medina D (2000). A mammary-specific model demonstrates the role of the p53 tumor suppressor gene in tumor development. Oncogene.

[31] Wang RA, Zhang H, Balasenthil S, Medina D, Kumar R (2006). PAK1 Hyperactivation is Sufficient for Mammary Gland Tumor Formation. Oncogene.

[32] Wilgenbus KK, Kirkpatrick CJ, Knuechel R, Willecke K, Traub O (1992). Expression of Cx26, Cx32 and Cx43 gap junction proteins in normal and neoplastic human tissues. Int J Cancer.

[33] Medina D (1974). Mammary tumorigenesis in chemical carcinogen-treated mice. II. Dependence on hormone stimulation for tumorigenesis. J Natl Cancer Inst.

[34] Christov K, Swanson SM, Guzman RC, Thordarson G, Jin E, Talamantes F, Nandi S (1993). Kinetics of mammary epithelial cell proliferation in pituitary isografted BALB/c mice. Carcinogenesis.

[35] Momiyama M, Omori Y, Ishizaki Y, Nishikawa Y, Tokairin T, Ogawa J, Enomoto K (2003). Connexin26-mediated gap junctional communication reverses the malignant phenotype of MCF-7 breast cancer cells. Cancer Sci.

[36] Zhang JT, Nicholson BJ (1994). The topological structure of connexin 26 and its distribution compared to connexin 32 in hepatic gap junctions. J Membr Biol.

[37] Laird DW (2010). The gap junction proteome and its relationship to disease. Trends Cell Biol.

[38] Henzl MT, Thalmann I, Larson JD, Ignatova EG, Thalmann R (2004). The cochlear F-box protein OCP1 associates with OCP2 and connexin 26. Hear Res.

[39] Chandrasekhar A, Kalmykov EA, Polusani SR, Mathis SA, Zucker SN, Nicholson BJ (2013). Intracellular redistribution of cAMP underlies selective suppression of cancer cell growth by connexin26. PLoS One.

[40] Mese G, Sellitto C, Li L, Wang HZ, Valiunas V, Richard G, Brink PR, White TW (2011). The Cx26-G45E mutation displays increased hemichannel activity in a mouse model of the lethal form of keratitis-ichthyosis-deafness syndrome. Mol Biol Cell.

[41] Schutz M, Auth T, Gehrt A, Bosen F, Korber I, Strenzke N, Moser T, Willecke K (2011). The connexin26 S17F mouse mutant represents a model for the human hereditary keratitis-ichthyosis-deafness syndrome. Hum Mol Genet.

[42] Kalra J, Shao Q, Qin H, Thomas T, Alaoui-Jamali MA, Laird DW (2006). Cx26 inhibits breast MDA-MB-435 cell tumorigenic properties by a gap junctional intercellular communication-independent mechanism. Carcinogenesis.

[43] Tan LW, Bianco T, Dobrovic A (2002). Variable promoter region CpG island methylation of the putative tumor suppressor gene Connexin 26 in breast cancer. Carcinogenesis.

[44] Kanczuga-Koda L, Sulkowski S, Tomaszewski J, Koda M, Sulkowska M, Przystupa W, Golaszewska J, Baltaziak M (2005). Connexins 26 and 43 correlate with Bak, but not with Bcl-2 protein in breast cancer. Oncol Rep.

[45] Conklin C, Huntsman D, Yorida E, Makretsov N, Turbin D, Bechberger JF, Sin WC, Naus CC (2007). Tissue microarray analysis of connexin expression and its prognostic significance in human breast cancer. Cancer Lett.

[46] Teleki I, Krenacs T, Szasz MA, Kulka J, Wichmann B, Leo C, Papassotiropoulos B, Riemenschnitter C, Moch H, Varga Z (2013). The potential prognostic value of connexin 26 and 46 expression in neoadjuvant-treated breast cancer. BMC Cancer.

[47] Teleki I, Szasz AM, Maros ME, Gyorffy B, Kulka J, Meggyeshazi N, Kiszner G, Balla P, Samu A, Krenacs T (2014). Correlations of differentially expressed gap junction connexins cx26, cx30, cx32, cx43 and cx46 with breast cancer progression and prognosis. PLoS One.

[48] Herschkowitz JI, Simin K, Weigman VJ, Mikaelian I, Usary J, Hu Z, Rasmussen KE, Jones LP, Assefnia S, Chandrasekharan S, Backlund MG, Yin Y, Khramtsov AI (2007). Identification of conserved gene expression features between murine mammary carcinoma models and human breast tumors. Genome Biol.

[49] Medina D (2007). Chemical carcinogenesis of rat and mouse mammary glands. Breast Dis.

[50] Vos CB, Cleton-Jansen AM, Berx G, de Leeuw WJ, ter Haar NT, van Roy F, Cornelisse CJ, Peterse JL, van de Vijver MJ (1997). E-Cadherin Inactivation in Lobular Carcinoma *in Situ* of the Breast: An Early Event in Tumorigenesis. Br J Cancer.

[51] Derksen PW, Braumuller TM, van der Burg E, Hornsveld M, Mesman E, Wesseling J, Krimpenfort P, Jonkers J (2011). Mammary-specific inactivation of E-cadherin and p53 impairs functional gland development and leads to pleomorphic invasive lobular carcinoma in mice. Dis Model Mech.

[52] Chao Y, Wu Q, Acquafondata M, Dhir R, Wells A (2012). Partial mesenchymal to epithelial reverting transition in breast and prostate cancer metastases. Cancer Microenviron.

[53] Stoletov K, Strnadel J, Zardouzian E, Momiyama M, Park FD, Kelber JA, Pizzo DP, Hoffman R, VandenBerg SR, Klemke RL (2013). Role of connexins in metastatic breast cancer and melanoma brain colonization. J Cell Sci.

[54] Kelly JJ, Simek J, Laird DW (2015). Mechanisms linking connexin mutations to human diseases. Cell Tissue Res.

[55] Avshalumova L, Fabrikant J, Koriakos A (2014). Overview of skin diseases linked to connexin gene mutations. Int J Dermatol.

[56] Coggshall K, Farsani T, Ruben B, McCalmont TH, Berger TG, Fox LP, Shinkai K (2013). Keratitis, ichthyosis, and deafness syndrome: a review of infectious and neoplastic complications. J Am Acad Dermatol.

[57] Caceres-Rios H, Tamayo-Sanchez L, Duran-Mckinster C, de la Luz Orozco M, Ruiz-Maldonado R (1996). Keratitis, ichthyosis, and deafness (KID syndrome): review of the literature and proposal of a new terminology. Pediatr Dermatol.

[58] Sakabe J, Yoshiki R, Sugita K, Haruyama S, Sawada Y, Kabashima R, Bito T, Nakamura M, Tokura Y (2012). Connexin 26 (GJB2) mutations in keratitis-ichthyosis-deafness syndrome presenting with squamous cell carcinoma. J Dermatol.

[59] Lydon J.P., Ge G., Kittrell F.S., Medina D., O'Malley B.W. (1999). Murine Mammary Gland Carcinogenesis is Critically Dependent on Progesterone Receptor Function. Cancer Res.

[60] Liebelt AG, Liebelt RA (1961). Effects of single pituitary isograft on mammary tumorigenesis in mice. Cancer Res.

[61] Plante I, Stewart MK, Laird DW (2011). Evaluation of mammary gland development and function in mouse models. J Vis Exp.

[62] Dunn T, Homburger F., Fishman N. H. (1959). Morphology of Mammary Tumors in Mice. Physiopathology of Cancer.

