# Supplementary material for: Cx26 knockout predisposes the mammary gland to primary mammary tumors in a DMBA-induced mouse model of breast cancer
Source: Oncotarget. 2015 Oct 2;6(35):37185–99. doi: 10.18632/oncotarget.5953 (PMC4741923; doi:10.18632/oncotarget.5953)
Supplement: Supplementary file 1 [file oncotarget-06-37185-s001.pdf]

Cx26 knockout predisposes the mammary gland to primary mammary tumors in a DMBA-induced mouse model of breast cancer

Supplementary Material

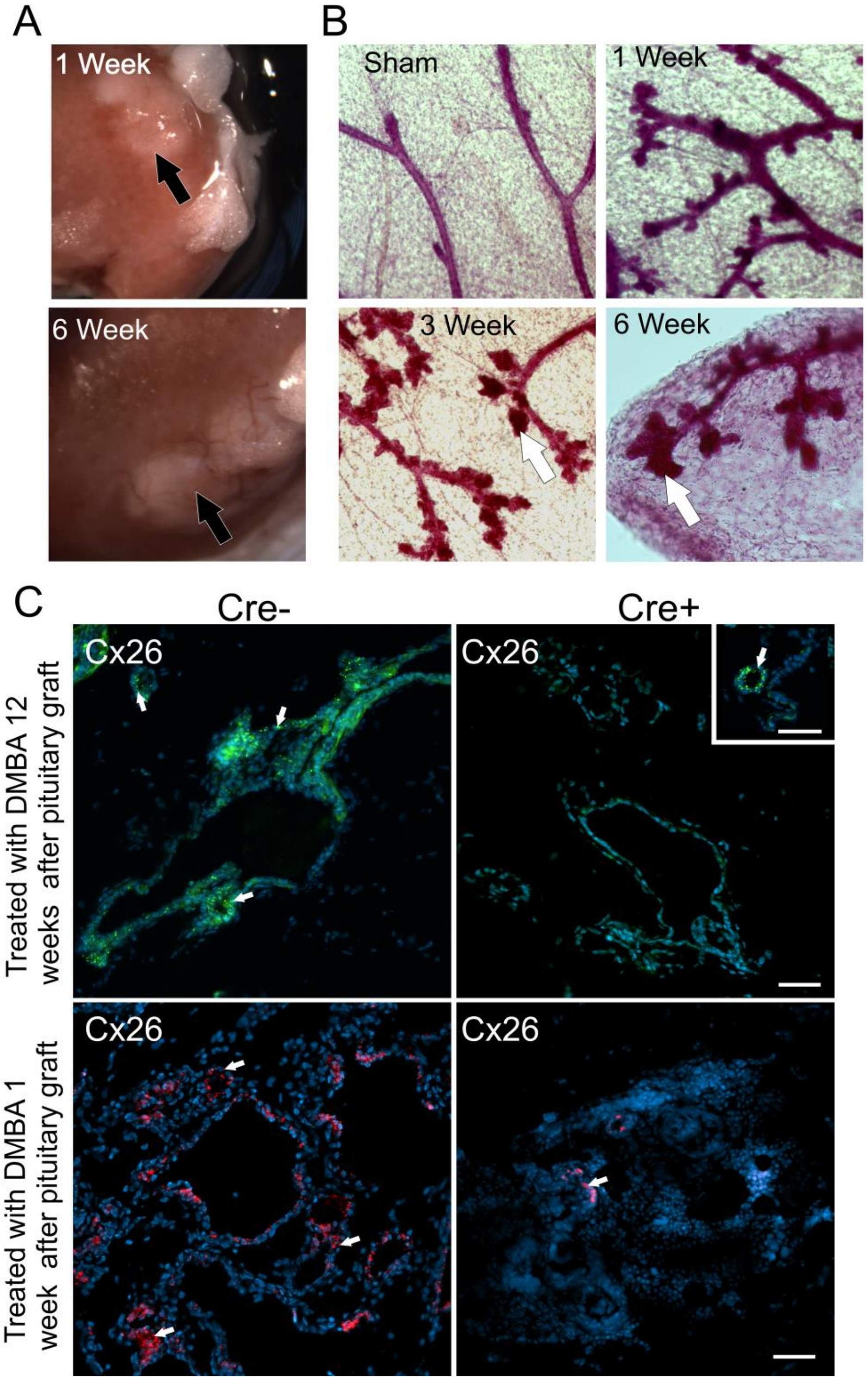

SFig 1. Pituitary implantation stimulates BLG that results in Cx26 knockout within the mammary gland. (A, B) Pituitaries were implanted in the renal capsule leading to the development of alveoli in the mammary gland which began to be seen 3 weeks following pituitary transplant but was more evident at 6 weeks. (C) At the time of sacrifice, immunofluorescence revealed considerable but not complete (insert) knockout of Cx26 (green/red, arrows) in the mammary gland of BLG-Cre; $Cx26^{fl/fl}$ mice (Cre+), unlike $Cx26^{fl/fl}$ control mice (Cre-) of both mice treated with DMBA 1 and 12 weeks after pituitary transplant. Hoechst denotes nuclei. Scale bars = 50 μm. N≥6.

STable1. List of Mice used in study for mice treated with DMBA or oil by gavage 12 weeks following pituitary transplant.

	Oil		DMBA	
Group 1<br>DMBA-treated Following Cx26 Knockdown	Cre-	Cre+	Cre-	Cre+
Initial N-Value For Study	7	7	16	12
# of Mice Removed from Study to date for other Health Concerns<br>- Lymphoma<br>- Intestinal Tumour<br>- Blood Clot<br>- Enlarged Lymph Node	0	0	4	3
Total # of Mice Included in Study	7	7	12	9
Total # of Mice with Mammary Tumours	0	0	4	8

STable2. List of Mice used in study for mice treated with DMBA or oil by gavage 1 week following pituitary transplant.

	Oil		DMBA	
Group 2<br>DMBA-Treated Prior to Cx26 Knockdown	Cre-	Cre+	Cre-	Cre+
Initial N-Value For Study	10	10	10	10
# of Mice Removed from Study to date for other Health Concerns<br>- Lymphoma<br>- Intestinal Tumour<br>- Unknown	2	2	3	3
Total # of Mice Included in Study	8	8	7	7
Total # of Mice with Mammary Tumours	0	0	7	7
